# Circular RNA circDVL1 inhibits clear cell renal cell carcinoma progression through the miR-412-3p/PCDH7 axis

**DOI:** 10.7150/ijbs.69351

**Published:** 2022-01-24

**Authors:** Ying Wang, Yunjing Zhang, Xinwan Su, Qiongzi Qiu, Yuan Yuan, Chunhua Weng, Sailan Zou, Yan Tian, Weidong Han, Pengyuan Liu, Xingyi Guo, Jianhua Mao, Xianghui Fu, Ping Wang, Weiqiang Lin

**Affiliations:** 1 The Fourth Affiliated Hospital, and International Institutes of Medicine, Zhejiang University School of Medicine, Jinhua 322000, Zhejiang, China.; 2 Kidney Disease Center, the First Affiliated Hospital, and Institute of Translational Medicine, Zhejiang University School of Medicine, Hangzhou 310003, Zhejiang, China.; 3 Department of Urology, the First Affiliated Hospital, Zhejiang University School of Medicine, Hangzhou 310003, Zhejiang, China.; 4 Department of Respiratory Medicine, Sir Run Run Shaw Hospital and Institute of Translational Medicine, Zhejiang University School of Medicine, Hangzhou 310016, Zhejiang, China.; 5 Division of Endocrinology and Metabolism, State Key Laboratory of Biotherapy and Cancer Center, West China Hospital, Sichuan University and Collaborative Innovation Center of Biotherapy, Chengdu 610041, Sichuan, China.; 6 Department of Medical Oncology, Sir Run Run Shaw Hospital, Zhejiang University School of Medicine, Hangzhou 310016, Zhejiang, China.; 7 Division of Epidemiology, Department of Medicine, Vanderbilt Epidemiology Center, and Vanderbilt-Ingram Cancer Center, Vanderbilt University School of Medicine, Nashville 37203, TN, USA.; 8 Department of Nephrology, National Clinical Research Center For Child Health, Children's Hospital, Zhejiang University School of Medicine, Hangzhou 310003, Zhejiang, China.

**Keywords:** circDVL1, Renal cell carcinoma, miR-412-3p, PCDH7, biomarker

## Abstract

Clear cell renal cell carcinoma (ccRCC) is a primary kidney cancer with high aggressive phenotype and extremely poor prognosis. Accumulating evidence suggests that circular RNAs (circRNAs) play pivotal roles in the occurrence and development of various human cancers. However, the expression, clinical significance and regulatory role of circRNAs in ccRCC remain largely unclear. Here we report that circDVL1 to be reduced in the serums and tissues from ccRCC patients, and to negatively correlate with ccRCC malignant features. Overexpression of circDVL1 inhibits proliferation, induces G1/S arrest, triggers apoptosis, and reduces migration and invasion in different ccRCC cells *in vitro*. Correspondingly, circDVL1 overexpression suppresses ccRCC tumorigenicity in a mouse xenograft model. Mechanistically, circDVL1 serves as a sponge for oncogenic miR-412-3p, thereby preventing miR-412-3p-mediated repression of its target protocadherin 7 (PCDH7) in ccRCC cells. Collectively, our results demonstrate that circDVL1 exerts tumor-suppressive function during ccRCC progression through circDVL1/miR-412-3p/PCDH7 axis, and suggest that circDVL1 could be a novel diagnostic and prognositc marker and therapeutic target for ccRCC.

## Introduction

Renal cell carcinoma (RCC) is an extremely lethal malignant carcinoma of the urological system and severely impacts human health 1. RCC incidence has increased in recent decades, with 73,750 new cases diagnosed in the United States in 2020 2, 3. Clear cell RCC (ccRCC), the most common and aggressive subtype of RCC, accounts for 70-75% of RCC cases 4. Importantly, more than 30% of ccRCC patients are associated with metastasis at initial diagnosis. Moreover, although radical surgery is the main treatment option for primary ccRCC, still around 40% of patients experienced relapse and metastases with 5-year overall survival of less than 10% 5, 6. These dilemmas underscore the urgent need to explore the molecular mechanisms underlying the development of ccRCC, both as diagnostic and prognostic biomarkers and as potential therapeutic targets.

Circular RNAs (circRNAs) are non-coding RNAs with single-chain closed-loop structures that make them highly stable 7, 8. Recent studies have demonstrated that circRNAs are abundant in blood 9, 10, saliva 11, and exosomes 12, indicating that they could serve as novel diagnostic biomarkers 13. Given that liquid biopsy is less invasive than traditional tissue biopsy 14, circRNAs have been considered valuable predictive tools for precision medicine 15-17. Specifically, several circRNAs, such as cricNOX4, circEGLN3 and circPRRC2A, may act as potential biomarkers for RCC diagnosis and/or prognosis, which have been summarized in our recent review 18. In addition, current research mainly focuses on the alternation of circRNA expression in ccRCC tissues 19, 20, however, their levels in liquid biopsy remain largely unknown, which hinders the exploration of potential biomarkers. Furthermore, although several circRNAs, including circHIAT1 21, circ-AKT3 22, and cRAPGEF5 23, have been shown to play important roles in the initiation and progression of ccRCC 18, systematic investigation of circRNAs implicated in ccRCC development, as well as their underlying molecular mechanisms, are still lacking.

Here, we first determined the expression of circRNAs in serum samples from ccRCC patients and healthy controls using a circRNA microarray. Significantly, we identified a novel ccRCC-associated circRNA circDVL1 (circBase ID: hsa_circ_ 0009267), which is encoded in the *DVL1* gene. CircDVL1 is significantly reduced in serum from ccRCC patients, and its expression is inversely correlated with ccRCC Fuhrman grading, suggesting the potential as a biomarker. Moreover, CircDVL1 is decreased in ccRCC tumor tissues. Correspondingly, circDVL1 can inhibit proliferation, induce cell cycle arrest, trigger apoptosis, and reduce ccRCC cell migration and invasion. Mechanistically, circDVL1 functions as a miR-412-3p sponge to upregulate PCDH7 expression. Collectively, these findings identify circDVL1 as a tumor suppressor for ccRCC and suggest its potential as both a diagnostic biomarker and a therapeutic target.

## Materials and Methods

### Clinical specimens

This study was approved by the Ethics Committee of the First Affiliated Hospital of Zhejiang University (2021-104). Eighteen matched samples of ccRCC and adjacent non-tumor tissues were collected from ccRCC patients. The specimens were quickly preserved in liquid nitrogen after the surgery and then stored at -80 °C until further use. Serum specimens from 60 ccRCC patients and 66 healthy controls were collected. Then, the samples were processed using appropriate methods, and the obtained serum was stored at -80 °C until further use.

### Cell lines and cell culture

All cell lines were purchased from the National Collection of Authenticated Cell Cultures (China). 786-O and Caki-1 cells were cultured in RPMI 1640 (HyClone), ACHN cells were cultured in MEM (HyClone), and 293T cells were cultured in DMEM (Gibco) media. All cell culture media were supplemented with 10% fetal bovine serum (Gibco) and 1% penicillin and streptomycin mixture (Gibco). Cell cultures were maintained in a humidified incubator at 37 °C and 5% CO_2_.

### CircRNA microarray analysis

CircRNA microarray profiling in serum samples was performed using an Arraystar human CircRNA Array V2 (Capital Biotechnology, China). GeneSpring software V13.0 (Agilent Technologies) was used for data summarization, normalization, and microarray quality control. CircRNAs with threshold pf fold change of ≥ 2 and ≤ -2 and *P*-value < 0.05 were regarded as statistically significant. Hierarchical cluster analysis was performed using standardized Euclidean distance with complete linkage.

### RNA sequencing (RNA-seq) and data analysis

Next-generation RNA-seq was carried out as detailed previously 24, 25. miR-412-3p and control mimic-transfected 786-0 cells were used for RNA sequencing. Library construction and bioinformatics analyses of the raw data were conducted by Novogene Co., Ltd. (Beijing, China). The RNA-seq data were deposited in Gene Expression Omnibus (GEO) under accession number GSE175492.

### RNA extraction and real-time quantitative RT-PCR (RT-qPCR)

RT-qPCR was performed as described previously 26, 27. Total RNA was isolated from serum, cells, and tumor tissues using AG RNAex Pro Reagent (AG, Hunan, China). For circRNA detection, 1 μg total RNA was digested with RNase R (3 U/μg, Epicenter Technologies, Madison, USA, Cat#RNR07250) at 37 °C for 10 min, and complementary DNA (cDNA) was generated using the HiScript II 1st Strand cDNA Synthesis Kit (Vazyme). CircRNA and mRNA expression levels were normalized to β-actin. To determine miRNA expression, reverse transcription was carried out with a miR-X^TM^ miRNA First-strand Synthesis kit (Takara Bio USA, CA, USA, Cat. #638313) with U6 (Rnu6-1) expression as an endogenous control. The relative RNA expression level was determined with the 2^-ΔΔCt^ method. All qRT-PCR analysis primers sequences are shown in Table S2.

### Plasmid construction and stable transfection

To construct the circDVL1 and circDVL1-MUT overexpression vector, the circDVL1 and mutated circDVL1 sequence were cloned into the pcDNA 3.1(+) Laccase 2 MCS Exon vector, which was ordered from Addgene (#69893) 28. ACHN and 786-O cells were transfected with circRNA and negative control vectors using Lipofectamine 3000 (Invitrogen, Carlsbad, CA). After 48 h, stably transfected cells expressing circDVL1 were selected by addition of 800 μg/mL G418 to the culture medium.

### Cell viability

Cell viability assay was determined with the CCK8 kit (Dojindo Laboratories, Kumamoto, Japan, Cat# CK04), as previously described 29. 786-O and ACHN circDVL1 overexpressing and control cells were seeded in 96-well plates. CCK8 solution (10 μL) was added at the indicated time and incubated at 37 °C for 2 h, and the absorbance was measured using a spectrophotometer.

### Colony formation

Colony formation assays were conducted as described previously 24. ACHN (800 cells/well) and 786-O cells (400 cells/well) were cultured for 10 days and subsequently fixed with 4% paraformaldehyde for approximately 30 min, followed by staining with 0.1% crystal violet solution for approximately 15 min. Clones containing more than 50 cells were counted as a single colony.

### Cell cycle analysis

Cells were synchronized in RPMI 1640 medium without FBS overnight and switched to medium with 10% FBS for the next 48 h. The cells were washed, re-suspended in 70% ethanol in PBS, and stored at -20 °C overnight. Next, cells were stained propidium iodide (PI) solution containing RNase A for cell cycle analysis.

### Apoptosis analysis

ACHN and 786-O cell apoptosis analysis was conducted with an Annexin V-FITC/PI apoptosis detection kit (Beijing Biosea Biotechnology, China) following the manufacturer's instructions.

### Cell migration and invasion assays

*In vitro,* cell migration capacity was evaluated using transwell chambers with 8 μm pore polycarbonate membranes (Millipore, MA, USA). Cell invasion assays were performed with the transwell chambers coated with 100 μL of Matrigel (BD Biosciences, USA). 786-O cells (2×10^4^) or ACHN cells (4×10^4^) were suspended in 200 μL of RPMI 1640 without serum and seeded into the upper transwell chambers, and then 600 μL of medium containing 20% FBS was added into the bottom chambers. Then, cells were cultured for 24 h to measure the migration and for 48 h to evaluate the invasion. Cells on the upper chamber were carefully scraped, and cells in the bottom compartment were fixed with 4% paraformaldehyde and stained with crystal violet. Images were obtained and cells were counted with a microscope.

### Western blot

Total protein was extracted, quantified, and separated on 10% SDS-PAGE gels, and then transferred to polyvinylidene difluoride membranes (Millipore, MA, USA), which were incubated with primary anti-glyceraldehyde 3-phosphate dehydrogenase (GAPDH) (Proteinch, 60004-1-Ig), anti-PCDH7 (Abcam, ab139274) primary antibodies at 4 °C overnight.

### Immunofluorescence staining

Cells were seeded onto coverslips for 24 h and fixed with 4% paraformaldehyde, permeabilized with 0.2% Triton X-100 for 20 min, and incubated with 5% bovine serum albumin. Next, cells were incubated with a primary antibody specific for Ki67 (1:400, ab48027, Abcam) at 4 °C overnight, and subsequently incubated with a 488-conjugated goat anti-rabbit IgG (1:150, D110088, Sangon) at 37 °C for 1 h. The cells were stained with 4′,6-diamidino-2-phenylindole (DAPI), and images were obtained with a Leica fluorescence microscope (DM4000).

### *In vivo* tumor formation and Ki67 staining

Six-week-old male BALB/c nude mice (purchased from Shanghai SLAC) were divided into 2 groups (n = 5 for each group). Mice were inoculated with approximately 3×10^6^ control cells or circDVL1 overexpressing ACHN cells by subcutaneous injection. Tumor size was measured with a sliding caliper every 3 days, starting day 6 after the injection. Tumor volume was calculated as length × width × height/2. All experiments were conducted according to the Guidelines for the Care and Use of Laboratory Animals (NIH publication 80-23, revised 1996) and were approved by the Animal Care Committee of Zhejiang University, Hangzhou, China.

### Fluorescence *in situ* hybridization (FISH)

A circDVL1 double-digoxigenin (DIG)-horseradish peroxidase (HRP) probe (Exon Biotechnology, Guangzhou, China) was synthesized. Control and circDVL1 overexpressing ACHN cells were cultured on cover slides and incubated with the labeled probe at 37 °C for 24 h. After washing, the slides were incubated in an anti-DIG secondary antibody at 37 °C for 1 h. A tyramine signal amplification method was used to detect circDVL1 signals, and cells were counterstained with DAPI. For co-FISH, cover slides were incubated with DIG-labeled circDVL1 and biotin-labeled miR-412-3p.

### RNA immunoprecipitation (RIP) assay

RIP assays were performed with the Magna RIP™ RNA-Binding Protein Immunoprecipitation Kit (Millipore, MA, USA). 786-O cells were lysed in complete RIP lysis buffer and then incubated with immunoprecipitation buffer containing magnetic beads which were conjugated with AGO2 (Abcam, MA, USA) or IgG antibody complex at 4 °C overnight. Then, immunoprecipitated RNAs were tested by RT-qPCR.

### Dual-luciferase reporter assay

CircDVL1, PPM1H-3'UTR, PCDH7-3'UTR sequences, which contained mutated miR-412-3p binding sites, and their corresponding mutated sequences, were synthesized and then subcloned into the luciferase reporter vector psiCHECK-2 (Promega) to produce the circDVL1-WT, circDVL1-Mut1, circDVL1-Mut2, PPM1H-3'UTR-WT, PCDH7-3'UTR-WT, PCDH7-3'UTR-Mut1, and PCDH7-3'UTR-Mut2 vectors. The different 3'UTR sequences of PPM1H and PCDH7 contain the 500 bp fragment containing potential miR-412-3p binding sites (flanked by 250 bp). The relative luciferase activity was assessed with a dual-luciferase reporter assay (Promega, USA). All assays were carried out in three independent replicates.

### Statistical analysis

Statistical analyses were conducted with GraphPad Prism 7.0 (GraphPad Software, La Jolla, CA). The differences between groups were assessed with Student's t-tests or one-way analysis of variance (ANOVA). Differences between groups were considered to be statistically significant when *P*-values were < 0.05.

## Results

### CircRNA expression profile and characterization of circDVL1 in ccRCC

**Figure 1:**
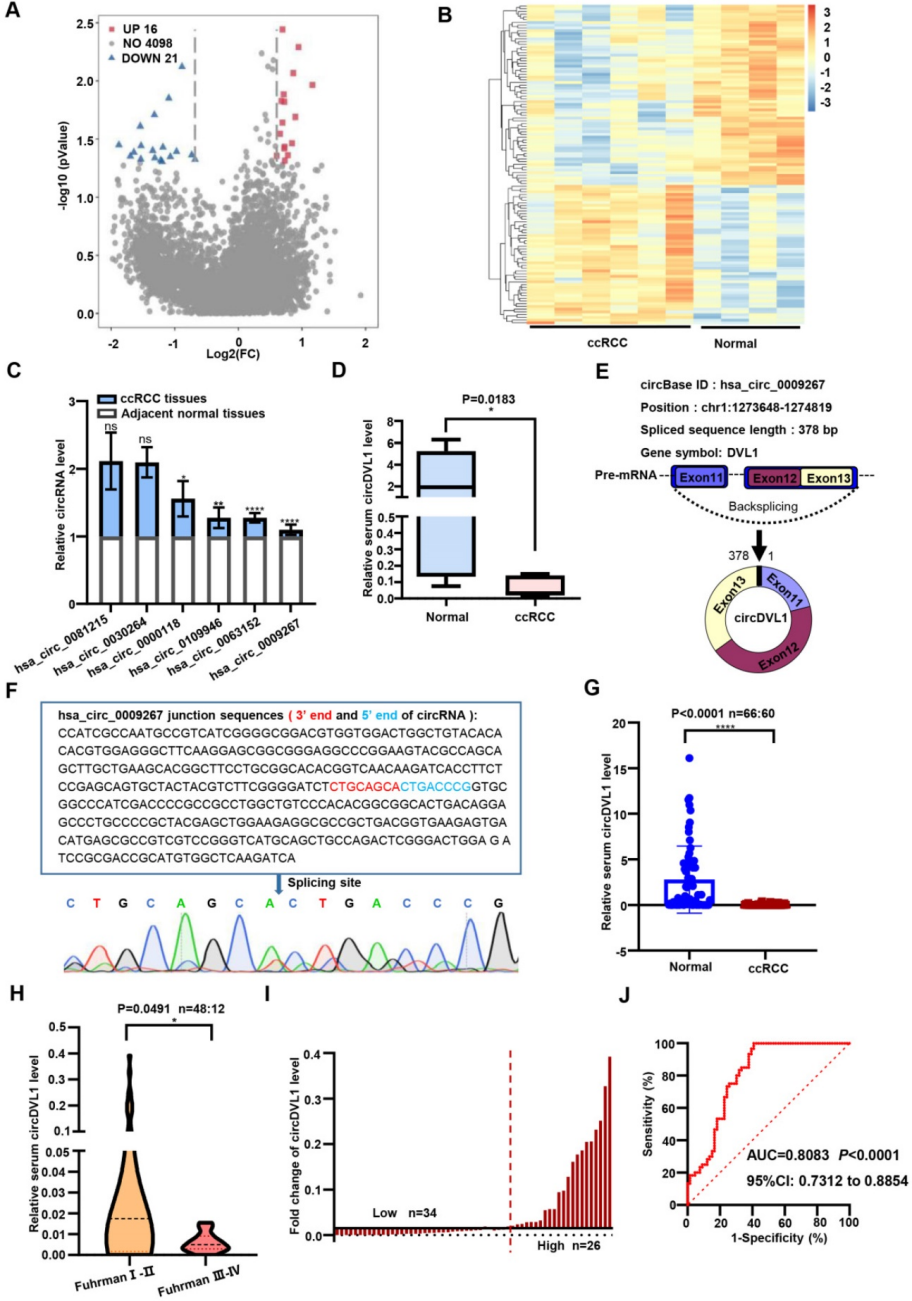
** CircDVL1 is reduced in the serum of ccRCC and could act as a potential biomarker for ccRCC diagnosis and prognosis. (A)** Volcano plot comparing the expression fold alterations of circRNAs in serum collected from ccRCC patients (n = 6) or healthy controls (n = 4). The circRNAs marked in blue and red indicate greater than two-fold changes between the different groups. **(B)** Heatmap of the clustered for differentially expressed circRNAs in ccRCC (P< 0.05), with rows representing circRNAs and columns representing serum samples. **(C)** RT-qPCR measured the expression of six circRNAs in 3 paired ccRCC and adjacent tissue samples. **(D)** Serum circDVL1 expression in ccRCC patients (n = 8) and healthy volunteers (n = 6) was determined by RT-qPCR. **(E)** The exonic information of circDVL1 (circBase ID: hsa_circ_0009267) is schematic as indicated. **(F)** The existence of circDVL1 was determined by Sanger sequencing; the blue arrow shows the back splice site. **(G)** RT-qPCR analysis the expression of serum circDVL1 in ccRCC patients (n = 60) and healthy volunteers (n = 66). **(H)** RT-qPCR analysis of serum circDVL1 expression in ccRCC Fuhrman I-II (n = 48), ccRCC Fuhrman III-IV patients (n=12). **(I)** Expression of circDVL1 in 60 patients with ccRCC serum. **(J)** ROC curve and AUC values for circDVL1 in ccRCC patients and healthy controls. Data are exhibited as mean ± SD. *P < 0.05, ** *P* < 0.01, *** *P* < 0.001, **** *P* < 0.0001.

As shown in Supplementary Fig. S1, our study was conducted in three phases, namely the discovery phase, training phase, and validation phase. To investigate the circRNA expression profile of ccRCC, we conducted a circRNA microarray (targeting 4,135 circRNAs) in serum samples from six ccRCC patients and four healthy controls. This analysis showed that 37 circRNAs were significantly differentially expressed at a threshold of nominal *P* < 0.05 and fold-change (FC) of ≥2.0 (Fig. 1A). Of these, 21 circRNAs were down-regulated in ccRCC patients compared with healthy controls, while the remaining 16 circRNAs were up-regulated (Fig. 1B). By screening the results, we selected the 18 most significantly differentially expressed circRNAs for further investigation. Initially, we designed divergent PCR primers to amplify these candidate circRNAs, and their products were verified through agarose gel electrophoresis. PCR results showed that the product sequences of hsa_circ_0030264, hsa_circ_0000118, hsa_circ_0081215, hsa_circ_0109946, hsa_circ_0009267 and hsa_circ_0063152 were singular (data not shown). To explore their implications in ccRCC progression, we evaluated the expression of these six circRNAs in three paired ccRCC samples using real-time quantitative PCR (RT-qPCR). Of the six circRNAs, hsa_circ_0009267 was ranked as the most differentially expressed circRNA (Fig. 1C) and thus was chosen for further investigation. Based on the annotation in circBase (http://www.circbase.org/), hsa_circ_0009267 is spliced from the *DVL1* gene located at chr1:1273648-1274819, and we named it circDVL1. To strengthen the potential clinical significance of circDVL1, we analyzed its expression in six normal serum samples and eight samples from ccRCC patients and found that circDVL1 expression was markedly decreased in ccRCC patients (Fig. 1D, *P* = 0.0183).

According to the annotation in circBase, circDVL1, 378 bp in length, is derived and cyclized from exons 11, 12, and 13 of *DVL1* (Fig. 1E) 30. To confirm the circular structure characteristics of circDVL1, we sequenced the PCR products of circDVL1 derived from ccRCC tumor samples. The results demonstrated the head-to-tail junction part of cirDVL1 (Fig. 1F). Hence, we further evaluated the clinical significance of circDVL1 in the validation phase.

### CircDVL1 acts as a potential serum diagnostic marker for ccRCC

We then examined serum circDVL1 levels in a validation cohort of 66 healthy participants and 60 ccRCC patients using RT-qPCR. Statistical analysis revealed that circDVL1 expression in serum from ccRCC patients was lower than in that of healthy participants (Fig. 1G, *P* < 0.0001). In addition, we grouped the ccRCC samples according to Fuhrman grading and determined the correlation with circDVL1 levels. These analyses showed that circDVL1 levels were decreased in Fuhrman grade III-IV ccRCC samples (n = 12) compared to Fuhrman grade I-II ccRCC samples (n = 48) (Fig. 1H, *P* = 0.0491). These results indicated that the expression level of circDVL1 is reversely associated with Fuhrman grade, suggesting that circDVL could be a potential diagnostic marker for ccRCC.

To determine the clinical implications of circDVL1 expression, we analyzed the association between circDVL1 expression in serum and the clinicopathological characteristics of ccRCC patients. Based on the levels of circDVL1 expression determined by RT-qPCR, 60 ccRCC patients were divided into circDVL1 low expression (n = 34) and circDVL1 high expression groups (n = 26) (Fig. 1I). The clinicopathological features of these patients are shown in Table S1. Statistical analysis showed that circDVL1 expression levels in serum were associated with Fuhrman grade (*P* = 0.05) (Table S1).

Receiver operating characteristic (ROC) curves and the corresponding area under the ROC curve (AUC) were used to evaluate the sensitivity and specificity of circDVL1 expression for ccRCC patient diagnosis by comparing cicrDVL1 expression in serum from ccRCC patients and healthy participants. The AUC value for circDVL1 expression in serum was 0.8083 (Fig. 1J). Therefore, circDVL1 could be used in potential diagnostic and prognostic applications.

### CircDVL1 inhibits ccRCC cell proliferation and induces apoptosis *in vitro*

**Figure 2:**
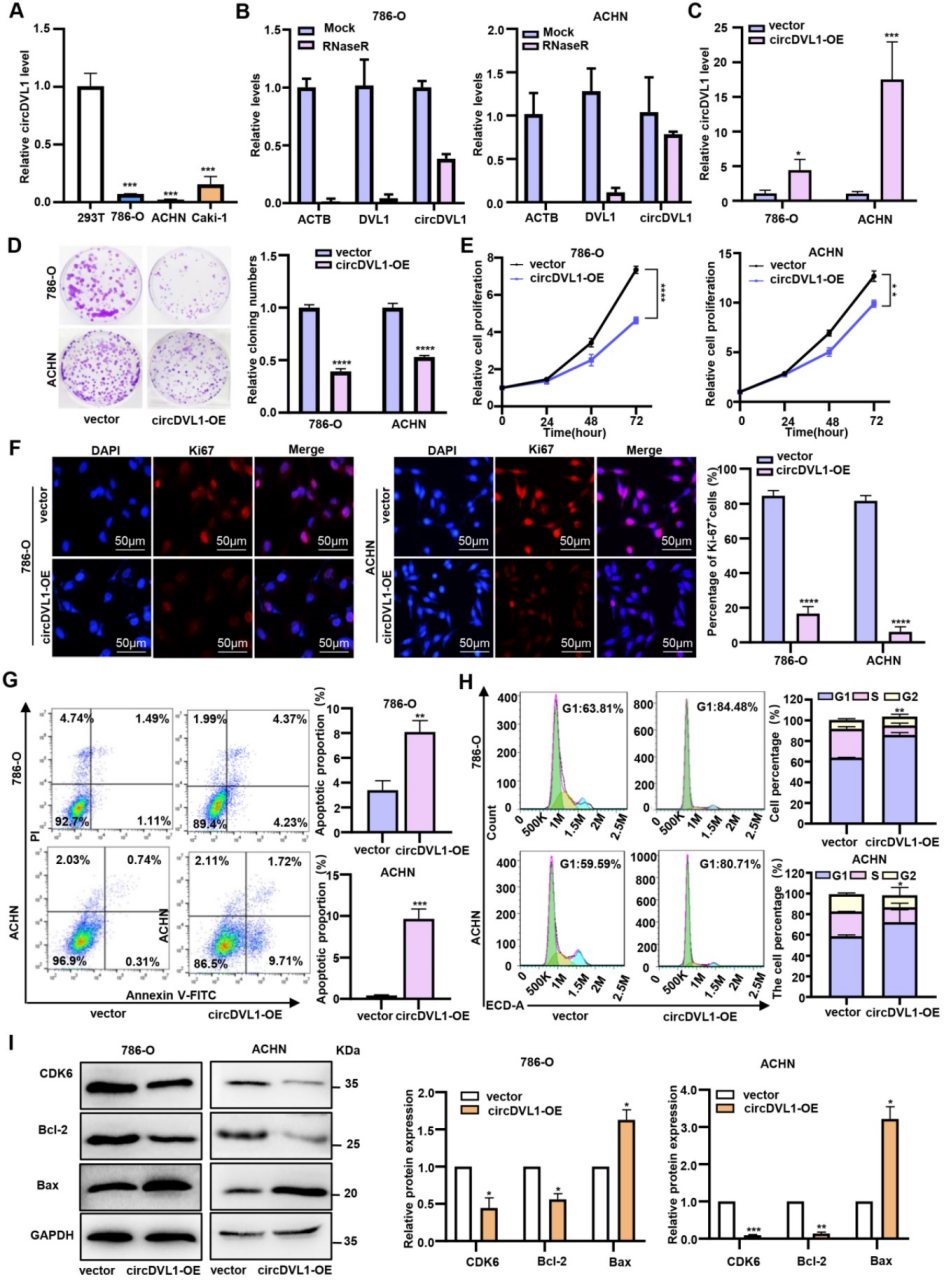
** CircDVL1 inhibits ccRCC cell proliferation and induces apoptosis *in vitro*. (A)** The expression of circDVL1 in several ccRCC cell lines and 293T cells were detected by RT-qPCR. **(B)** CircDVL1 was tolerant to RNase R. Linear RNAs β-actin (ACTB) and DVL1 were significantly decreased with RNase R, while circDVL1 was almost unaffected. Mock, absence of RNase R. **(C)** The abundance of circDVL1 in 786-O and ACHN cells transfected with circDVL1 and control vectors (Vector) was determined by RT-qPCR. **(D)** Colony formation of 786-O and ACHN transfected with circDVL1 overexpression (circDVL1-OE) or control vectors. **(E)** The proliferation ability of 786-O and ACHN transfected with circDVL1 overexpression or control vectors was assessed with the CCK-8 assay. **(F)** Ki67 expression of 786-O and ACHN transfected with circDVL1 overexpression or control vectors was assessed with immunofluorescence staining assays. Scale bars: 50 µm. **(G)** The percentage of apoptosis distributions cells in each stage was evaluated by flow cytometry. **(H)** Cell cycle profile of 786-O and ACHN transfected with circDVL1 overexpression or control vectors and quantitative analysis of results from two independent experiments are shown. **(I)** Western blot results of CDK6, Bcl-2, and Bax levels in 786-O and ACHN transfected with circDVL1 overexpression or control vectors, and quantitative analysis of results from two independent experiments are shown. GAPDH expression was served as a loading control. Data are exhibited as mean ± SD. *P < 0.05, ** P < 0.01, *** *P* < 0.001, **** *P* < 0.0001, no significant (NS).

CircDVL1 level was significantly decreased in tumor tissue and serum from ccRCC patients, suggesting that restoring its expression may prevent ccRCC tumorigenesis. Compared with 293T cells, ccRCC cells, particularly 786-O and ACHN, had markedly low basal expression levels of circDVL1 (Fig. 2A). Therefore, ACHN and 786-O cells were used to explore the effect of circDVL1 overexpression on ccRCC. To facilitate circDVL1 detection, we added RNase R to isolated total RNAs since it can digest linear RNAs with free 3' ends, but has no effect on circRNAs. This assay illustrated that circDVL1 was resistant to RNase R treatment (Fig. 2B), confirming it as a circRNA. To investigate the function of circDVL1, we constructed a circDVL1 expression vector to overexpress circDVL1 in 786-O and ACHN cells. The level of circDVL1 was effectively upregulated in 786-O and ACHN cells transfected with the circDVL1 expression vector (Fig. 2C). Subsequently, clone formation, proliferation, and immunofluorescence assays were carried out to assess the effects of circDVL1 *in vitro*. circDVL1 overexpression resulted in decreased ccRCC cell colony numbers and reduced ccRCC cell survival ability (Fig. 2D). Furthermore, the CCK-8 proliferation assay revealed that circDVL1 overexpression significantly inhibited the proliferation of 786-O and ACHN cells (Fig. 2E). Interestingly, immunofluorescence assays showed a decreased Ki67 proliferation index in 786-O and ACHN cells overexpressing circDVL1 (Fig. 2F). These data demonstrated that circDVL1 overexpression inhibits ccRCC cell proliferation.

Next, we conducted flow cytometric analyses to assess whether circDVL1 expression could affect apoptosis of 786-O and ACHN cells. Overexpression of circDVL1 induced apoptosis in 786-O and ACHN cells (Fig. 2G). Moreover, flow cytometry analysis showed that circDVL1 overexpression triggered G0-G1 arrest in 786-O and ACHN cells (Fig. 2H). Correspondingly, circDVL1 overexpression was found to decrease the levels of the cell cycle regulator CDK6 and the apoptosis regulators Bcl-2 and Bax (Fig. 2I). Additionally, transwell assays revealed that circDVL1 overexpression significantly inhibited ccRCC cell migration and invasion (Supplementary Fig. S2). Together, these data showed that circDVL1 could induce G1/S arrest, trigger apoptosis, and reduce migration and invasion in ccRCC cells *in vitro*.

### CircDVL1 prevents the growth of ccRCC tumors *in vivo*

**Figure 3:**
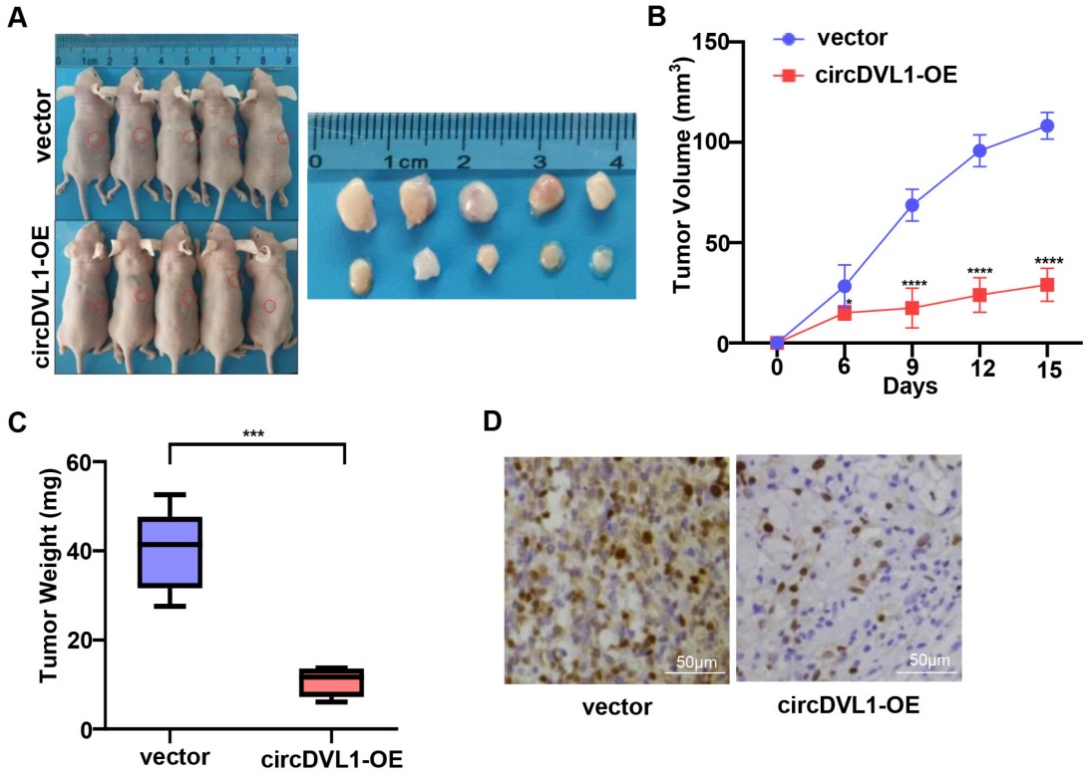
** CircDVL1 prevents the growth of ccRCC tumors *in vivo*. (A)** ACHN cells transfected with circDVL1 overexpression or control vectors were injected subcutaneously into nude mice (n = 5) as illustration. After 15 days, tumors of mice were dissected. **(B)** The volume of xenografted tumors in nude mice was calculated every 3 days. **(C)** Tumor growth was measured in the control vector and the circDVL1-overexpressing groups. Tumor weights were analyzed after removing the tumors. **(D)** IHC for Ki67 in the control vector and the circDVL1-overexpressing group. Positive cells showed distinct, brown nuclear staining. circDVL1-OE, circDVL1 overexpression. vector, negative control for overexpression. Data are exhibited as mean ± SD; *P < 0.05, ** *P* < 0.01, *** P < 0.001, **** *P* < 0.0001, no significant (NS).

To further explore the biological functions of circDVL1 *in vivo*, we developed tumor xenograft models in nude mice by subcutaneously injecting ACHN cells stably transfected with circDVL1 expression and control vectors (n = 5 for each group). After 15 days, we found that tumors in mice injected with circDVL1 overexpressing cells had smaller volumes and lower weights than those of the control mice (Fig. 3A-C), demonstrating that circDVL1 could inhibit ccRCC cell-derived tumor growth *in vivo*. Consistently, IHC analysis showed that Ki67 expression was decreased in circDVL1-expressing tumors compared to control tumors (Fig. 3D). These results demonstrated that overexpression of circDVL1 is sufficient to suppress ccRCC cell-derived tumor growth *in vivo*.

### CircDVL1 serves as a molecular sponge for miR-412-3p

**Figure 4:**
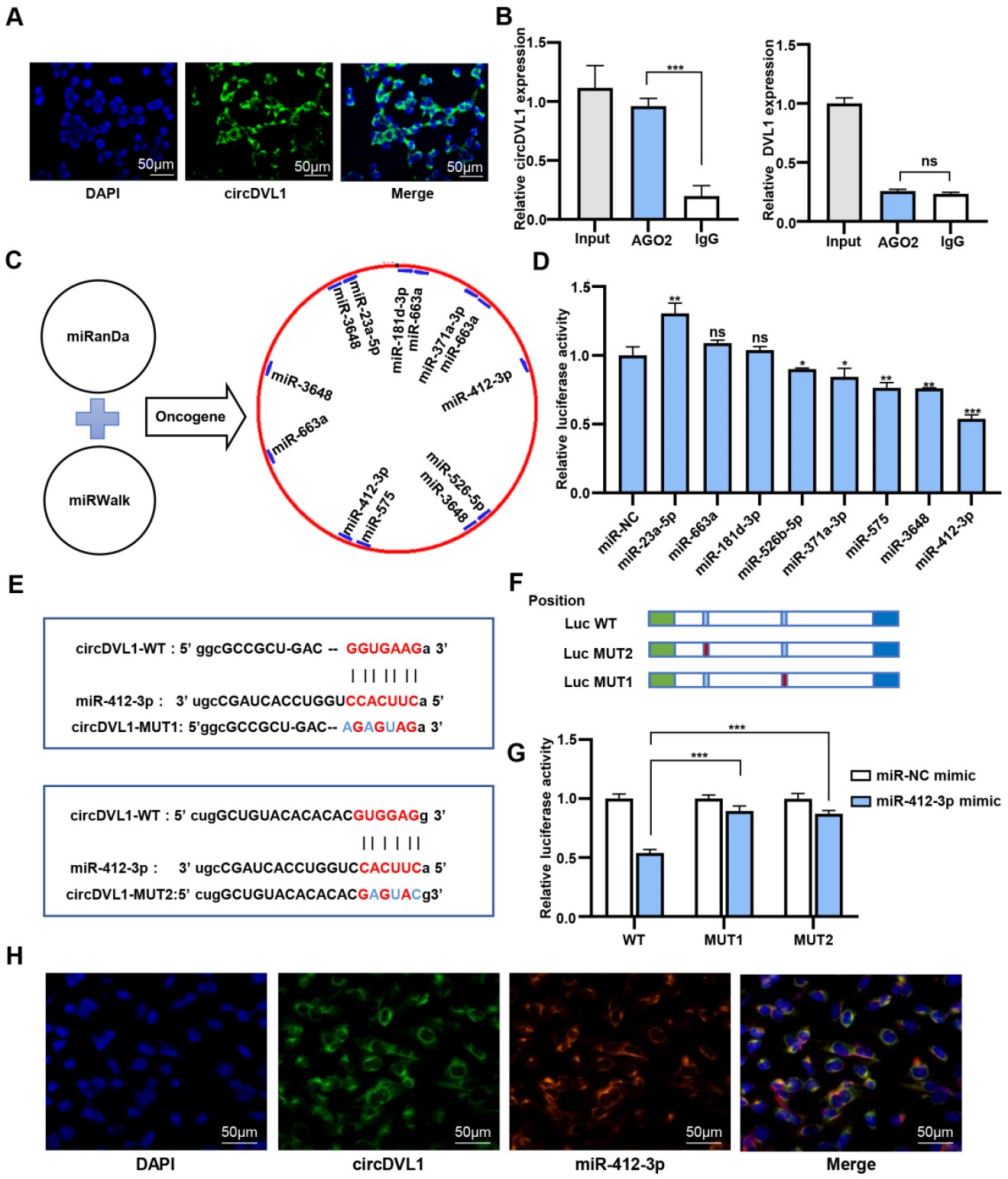
** CircDVL1 functions as a molecular sponge for miR-412-3p in ccRCC cells. (A)** FISH analysis showed that circDVL1 (green) and DAPI (blue) localized to the cytoplasm of 786-O. Scale bar: 50 µm. **(B)** RNA immunoprecipitation (RIP) and RT-qPCR assays to detect circDVL1 and DVL1 were immunoprecipitated by AGO2 and IgG antibodies in 786-O. **(C)** The schematic flowchart shows the pipelines that can detect oncogenic miRNA bound to circ-DVL1 3'-UTR via the online bioinformatics network. **(D)** Dual-luciferase reporter assays were used to evaluate candidate miRNAs associated with circDVL1 in 293T cells.** (E and F)** Sequence of Wild-type (WT) and mutated (MUT) putative miR-412-3p-binding sites in the 3'-UTR of circDVL1. **(G)** Relative luciferase activities were examined in 293T after transfection with circDVL1-WT or circDVL1-MUT and miR-412-3p mimic or miR-NC. **(H)** RNA CO-FISH assay for circDVL1 (green), miR-412-3p (red), DAPI (blue), and purple in merge in 786-O cells. miR-NC mimic was used as the miRNA mimic negative control. Scale bar, 100 µm. Data are presented as mean ± SD. *P < 0.05, ** *P* < 0.01, *** *P* < 0.001, **** *P* < 0.0001, no significant (NS).

To elucidate the circDVL1 regulatory mechanism in ccRCC cells, we performed fluorescence *in situ* hybridization (FISH) to investigate the subcellular localization of circDVL1. The FISH results demonstrated that circDVL1 was mainly localized in the cytoplasm (Fig. 4A), indicating that circDVL1 could be a miRNA sponge. To test this idea, we performed an anti-Ago2 immunoprecipitation assay in 786-O cells. RIP and RT-qPCR analysis demonstrated that endogenous circDVL1, but not DVL1, in 786-O cells was specifically enriched in Ago2-immunoprecipitates compared with IgG-control immunoprecipitates (Fig. 4B), supporting the idea that circDVL1 could bind to Ago2 and act as a miRNA sponge. Bioinformatics analysis using the MiRanda and miRWalk databases indicated that 132 potential miRNAs might interact with circDVL1. Eight potential oncogenic miRNAs were selected for further analysis, including miR-181d-3p, miR-575, miR-412-3p, miR-371a-3p, miR-663a, miR-526b-5p, miR-23a-5p, and miR-3648 (Fig. 4C). Then, we constructed wild-type psiCHECK-2 circDVL1 vectors containing binding sites for the identified miRNAs. Dual-luciferase reporter assays were carried out to evaluate the potential interaction between circDVL1 and these miRNA candidates. Among them, miR-412-3p mimics exhibited the most inhibitory effect on luciferase activity compared to the miRNA mimic negative control (Fig. 4D), and thus this miRNA was selected for further investigation.

To further confirm whether miR-412-3p could regulate circDVL1 expression, we developed circDVL1-miR-412-3p mutant1 (MUT1) and mutant2 (MUT2) vectors (Fig. 4E and F), and then cotransfected HEK 293 cells with these vectors and miR-412-3p or miR-NC mimics. The results revealed that miR-412-3p mimics repressed circDVL1-WT luciferase activity, whereas this inhibition was almost abolished in MUT1 and MUT2 expressing cells (Fig. 4G), suggesting a direct link between circDVL1 and miR-412-3p. miR-412-3p was significantly reduced in circDVL1-overexpressing 786-O and ACHN cells (Supplementary Fig. S3), and circDVL1 and miR-412-3p were primarily colocalized in the cytoplasm as shown by a co-FISH assay (Fig. 4H), further supporting the role of circDVL1 in miR-412-3p regulation. Collectively, these results showed that circDVL1 could serve as a molecular sponge for miR-412-3p thereby decreasing its expression.

### miR-412-3p promotes ccRCC cell proliferation, migration, and invasion *in vitro*

**Figure 5:**
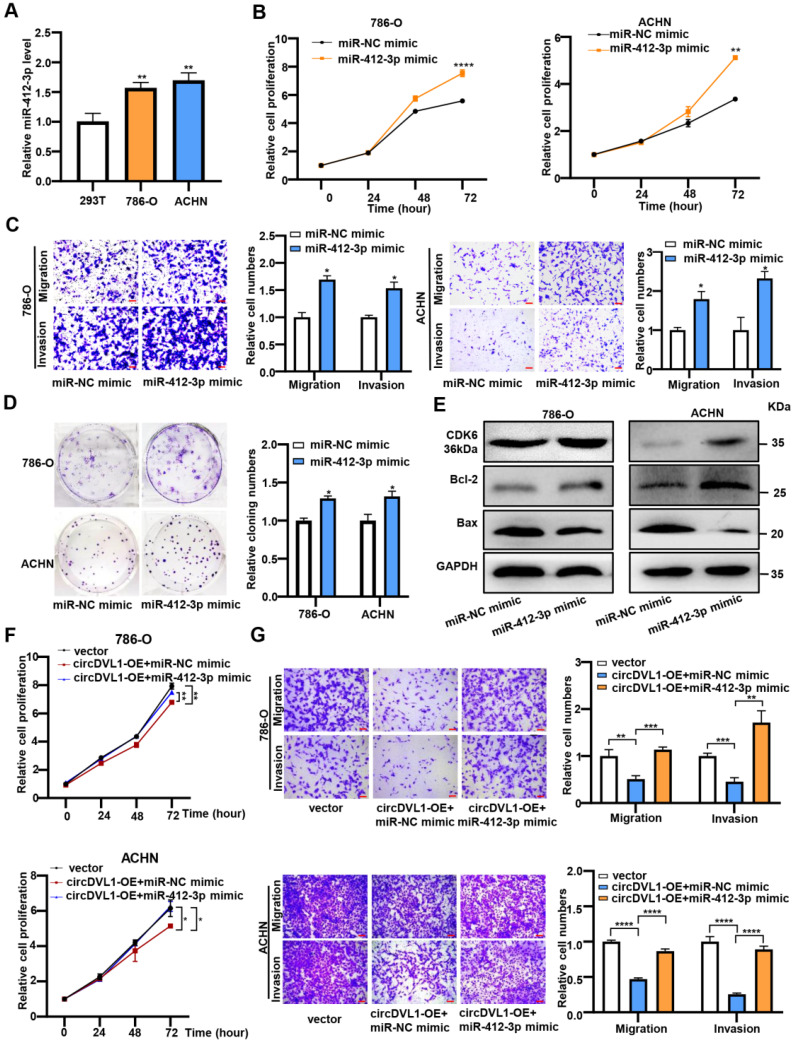
** miR-412-3p promotes ccRCC cell proliferation, migration and invasion *in vitro*. (A)** The expression levels of miR-412-3p in 786-O, ACHN, and 293T were detected by RT-qPCR. **(B)** The proliferation of 786-O and ACHN transfected with miR-412-3p mimic and miR-NC mimic was detected by the CCK-8 proliferation assay. **(C)** Transwell migration and invasion assays indicated that miR-412-3p mimic enhanced the migration and invasion capacities of 786-O and ACHN after transfection. **(D)** The colony formation ability of 786-O and ACHN transfected with miR-412-3p mimic and miR-NC. **(E)** Western blot results of CDK6, Bcl-2, and Bax levels in ccRCC cells lines after transfection with miR-412-3p mimic or miR-NC. **(F)** CCK-8 cell proliferation analysis of 786-O and ACHN cotransfected with control vectors + control mimics or circDVL1 overexpression vector + control mimics or circDVL1 overexpression vector + miR-412-3p mimics. **(G)** Transwell cell invasion and migration assay in treated 786-O and ACHN cells. Scale bar, 100 µm. circDVL1-OE, circDVL1 overexpression vector, negative control for overexpression. miR-NC mimic was used as the miRNA mimic negative control. Data are exhibited as mean ± SD. *P < 0.05, ** *P* < 0.01, *** *P* < 0.001, **** *P* < 0.0001, no significant (NS).

Having confirmed the link between circDVL1 and miR-412-3p, we further investigated the potential biological roles of miR-412-3p in ccRCC. It has been reported that miR-412-3p promoted colon cancer progression by enhancing cancer stem cell proliferation and metastasis 31. Firstly, we assessed the level of miR-412-3p in ccRCC cells and found that it was upregulated in ccRCC cells compared with 293T cells (Fig. 5A). Subsequently, CCK-8 proliferation, colony formation, and transwell assays showed that miR-412-3p mimics significantly enhanced proliferation, migration, and invasion of ccRCC cells (Fig. 5B-D). Moreover, western blot analysis was performed to determine the levels of proteins associated with the cell cycle and apoptosis. The results showed that 786-O and ACHN cells over-expressed with miR-412-3p had increased CDK6 and Bcl-2 levels and decreased Bax protein levels compared with control cells (Fig. 5E and Supplementary Fig. S4). Collectively, these results suggested that miR-412-3p could promote ccRCC tumor growth and progression.

Next, we cotransfected the circDVL1 overexpression vector and miR-412-3p mimics into ccRCC cells to test whether circDVL1 exerts its tumor-suppressive effects by sponging miR-412-3p. The results illustrated that miR-412-3p could partially reverse the circDVL1-mediated reduction in cell proliferation (Fig. 5F), invasion, and migration (Fig. 5G) in 786-O and ACHN cells. Next, we performed a rescue assay including four groups (vector, vector+miR-412-3p, miR-412-3p+circDVL1-OE, miR-412-3p+ circDVL1-MUT-OE). We found that circDVL1, but not MUT circDVL1, can still suppress tumor progression in the presence of miR-412-3p (Supplementary Fig. S5A and S5B). Overall, these results showed that circDVL1 inhibited ccRCC cell proliferation, invasion, and migration partly by regulating the function of miR-412-3p.

### PCDH7 is a target of miR-412-3p and suppresses ccRCC proliferation, migration, and invasion

**Figure 6:**
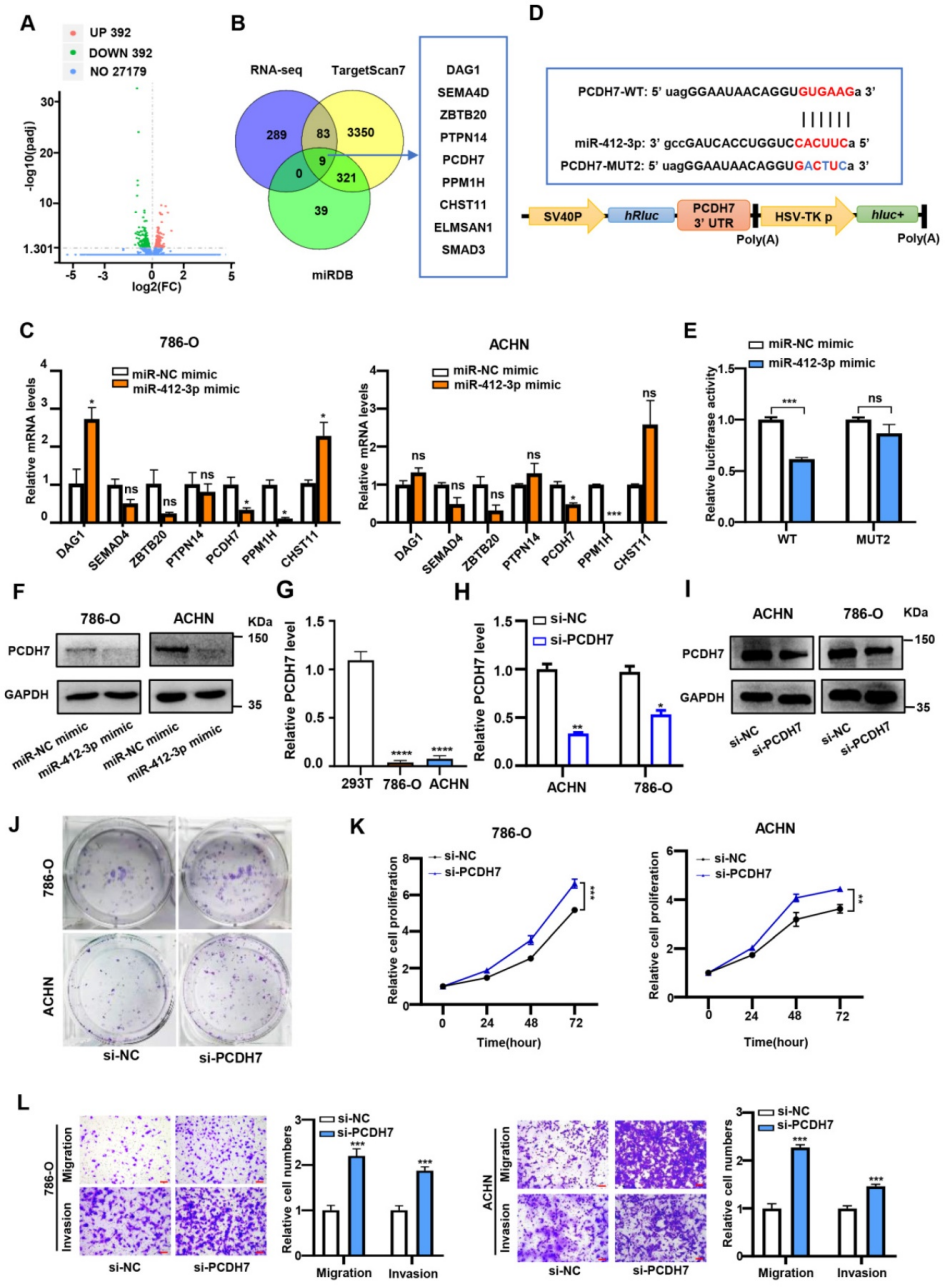
** PCDH7 is a target of miR-412-3p and suppresses ccRCC proliferation, migration and invasion. (A)** Volcano plot showing downstream genes with significantly altered expression in 786-O cell overexpressing miR-412-3p and miR-NC mimics. **(B)** Schematic illustration exhibiting the overlap between the target mRNAs and miR-412-3p as detected by TargetScan, miRDB, and RNA-seq. **(C)** 786-O and ACHN were transfected with miR-412-3p or miR-NC mimics for 48 h, and the expression of downstream genes was analyzed by qPCR. **(D)** The sequence of WT and MUT putative miR-412-3p-binding sites in the 3'-UTR of PCDH7. **(E)** Relative luciferase activities were examined in 293T after transfection with PCDH7-WT or PCDH7-MUT and miR-412-3p mimic or miR-NC. **(F)** Western blot results of PCDH7 levels in ccRCC cell lines after transfection with miR-412-3p mimic or miR-NC. **(G)** PCDH7 expression in 786-O, ACHN, and 293T was analyzed by RT-qPCR. **(H)** The relative abundance of PCDH7 in 786-O and ACHN cells transfected with si-NC and si-PCDH7 was determined by qRT-PCR. **(I)** Western blot of PCDH7 levels in ccRCC cell lines after transfection si-NC and si-PCDH7. **(J)** Colony formation ability of 786-O and ACHN cells transfected with si-NC and si-PCDH7. **(K)** The proliferation ability of 786-O and ACHN transfected with si-NC and si-PCDH7 was tested by the CCK-8 assay. **(L)** Transwell migration and invasion assays indicated that si-PCDH7 promoted migration and invasion capacity of transfected 786-O and ACHN. Scale bar, 100 µm. si-NC, siRNA for NC. si-PCDH7, siRNA against PCDH7. miR-NC mimic was used as the miRNA mimic NC. Data are exhibited as mean ± SD. *P < 0.05, ** *P* < 0.01, *** *P* < 0.001, **** *P* < 0.0001, no significant (NS).

To explore the signaling network involving circDVL1, miR-412-3p and its targets in ccRCC, we used RNA sequencing (RNA-seq) to identify potential miR-412-3p target genes in miR-412-3p overexpressing and miR-NC control cells. RNA-seq data identified 392 mRNAs that were downregulated in miR-412-3p overexpressing 786-O cells (Fig. 6A). We conducted bioinformatics analysis to identify predicted targets of miR-412-3p by using the TargetScan and miRDB databases. Venn analysis of RNA-seq data, TargetScan, and miRDB revealed nine overlapping genes: *SEMA4D*, *DAG1*, *PPM1H*, *ELMSAN1*, *ZBTB20*, *CHST11*, *PCDH7*, *PTPN14*, and *SMAD3* (Fig. 6B). We next transfected the miR-412-3p mimics into ccRCC cells and determined the expression levels of potential target genes by RT-qPCR. We found that *PPM1H* and *PCDH7* expression was significantly reduced in ccRCC cells transfected with miR-412-3p mimics (Fig. 6C). To further investigate the selected targets, we constructed luciferase reporter vectors driven by the wild-type (WT) 3'-UTR sequence of *PPM1H* and *PCDH7*, which contained potential miR-412-3p binding sites (PPM1H-WT and PCDH7-WT) (Fig. 6D and Supplementary Fig. S6A). HEK-293 cells were co-transfected with these plasmids and miR-412-3p or miR-NC mimics. We observed that the luciferase activity of PCDH7-WT, but not PPM1H-WT, was inhibited by miR-412-3p (Fig. 6E and Supplementary Fig. S6B). Furthermore, we found that miR-412-3p did not affect PCDH7-MUT luciferase activity (Fig. 6E). These data suggested that the 3'UTR of PCDH7 mRNA contained a miR-412-3p binding site. Western blot analysis revealed that miR-412-3p mimics markedly decreased PCDH7 protein levels (Fig. 6F and Supplementary Fig. S6C). We next evaluated PCDH7 mRNA level in an RNA-seq data set of ccRCC tumors from The Cancer Genome Atlas (TCGA) cohort. As shown in Supplementary Fig. S6D, the results demonstrated a decrease in PCDH7 mRNA in ccRCC tumors. Next, we evaluated the expression level of PCDH7 in ACHN and 786-O cells. The results revealed that PCDH7 was downregulated in ccRCC cells compared with 293T cells (Fig. 6G). Next, we transfected 786-O and ACHN cells with siRNAs targeting PCDH7 (si-PCDH7) and negative control siRNA (Fig. 6H and Fig. 6I) and then conducted proliferation, colony formation, and transwell assays. Colony formation assays revealed that PCDH7 knockdown enhanced ccRCC cell proliferation (Fig. 6G). CCK-8 proliferation assays indicated that PCDH7 silencing significantly promoted 786-O and ACHN cell proliferation (Fig. 6K). Further, transwell assays showed that PCDH7 silencing improved the migratory and invasion capability of 786-O and ACHN cells (Fig. 6L).

### CircDVL1 inhibits ccRCC progression via the miR-412a-3p-PCDH7 pathway

**Figure 7:**
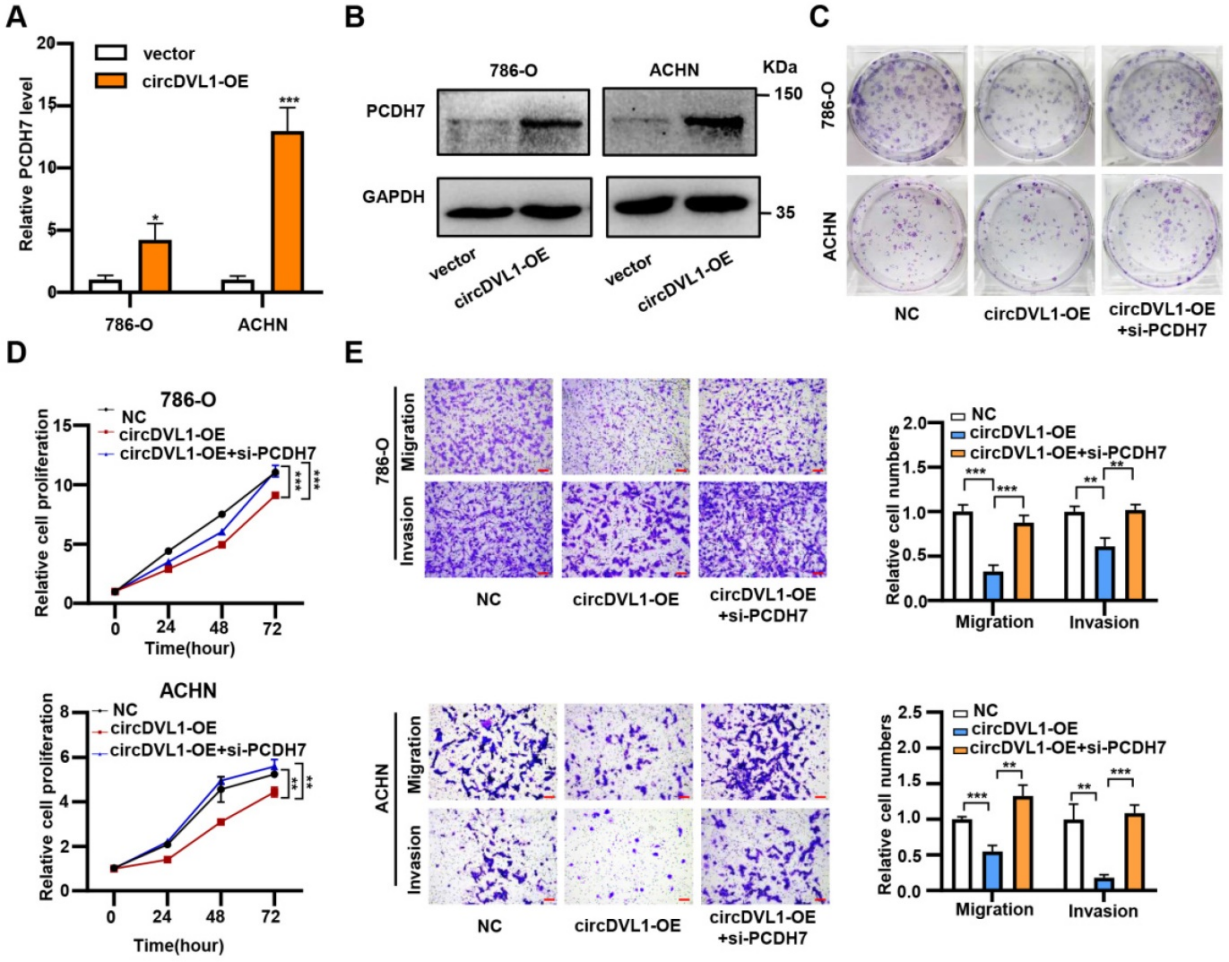
** CircDVL1 represses ccRCC progression via the miR-412a-3p-PCDH7 pathway. (A and B)** Relative mRNA and protein levels of PCDH7 were checked in 786-O and ACHN cells after transfected with control vector and circDVL1 overexpression vector using RT-qPCR and western blot. **(C and D)** Silencing of PCDH7 reversed circDVL1 overexpressing-induced cell proliferation and colony formation ability inhibition. **(E)** Silencing PCDH7 significantly reversed circDVL1 overexpressing-induced cell migration and invasion suppression. Scale bar, 100 µm. si-PCDH7, siRNA against PCDH7. circDVL1-OE, circDVL1 overexpression. NC, empty vector for overexpression. Data are exhibited as mean ± SD. *P < 0.05, ** *P* < 0.01, *** *P* < 0.001, **** *P* < 0.0001, no significant (NS).

In line with the regulation of circDVL1 on miR-412-3p, overexpression of circDVL1 significantly increased PCDH7 mRNA expression and protein levels (Fig. 7A, B and Supplementary Fig. S7). To investigate whether circDVL1 exerts its biological function by regulating PCDH7 expression, we performed a rescue assay. Proliferation and colony formation assays showed that PCDH7 silencing partially reversed circDVL1 suppression-induced growth inhibition in 786-O and ACHN cells (Fig. 7C and D). Similarly, PCDH7 downregulation partially reversed the circDVL1-mediated reduction in migration and invasion of ccRCC cells (Fig. 7E). Next, we performed a rescue assay including three groups (si-NC, si-PCDH7, si-PCDH7+circDVL1-OE). The analysis of proliferation and colony formation assays (**Figure S8A and S8B**) indicated an increase of the progression of ccRCC cells in PCDH7 knockdown cells can be partially suppressed by overexpression of circDVL1. Because PCDH7 knockdown only caused about 50-60% loss of PCDH7 mRNA level (Figure 6H), PCDH7 still existed in the knockdown cells and is regulated by circDVL1. Taken together, these results suggested that PCDH7, the downstream target of miR-412a-3p, partially mediates the suppressive roles of circDVL1 in ccRCC progression.

### CircDVL1 is decreased in human ccRCC tissues and correlates with miR-412a-3p and PCDH7

**Figure 8:**
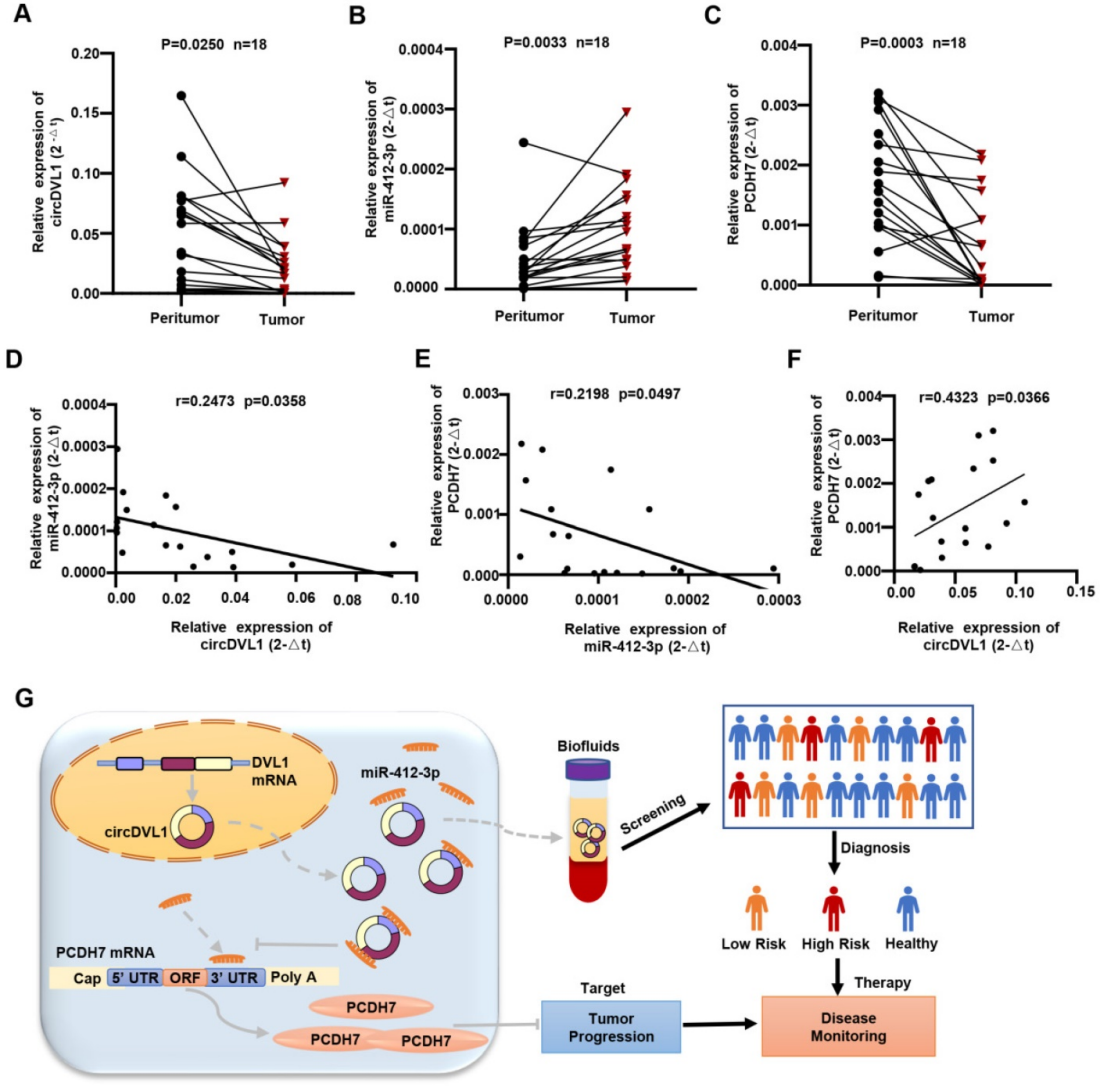
** CircDVL1 is decreased in human ccRCC tissues and correlates with miR-412a-3p and PCDH7. (A)** The expression of circDVL1 in 18 matched ccRCC and adjacent normal tissue samples was tested by RT-qPCR. **(B)** The expression of miR-412-3p was carried out by performing RT-qPCR assays in 18 matched ccRCC tumors and adjacent normal tissue. **(C)** The levels of PCDH7 in ccRCC tumor and adjacent normal tissues were measured using RT-qPCR (n = 18). **(D-F)** Pearson correlation analysis between circDVL1, miR-412-3p and PCDH7 expression in 18 ccRCC samples. **(G)** Schematic illustration of the mechanism through which circDVL1 inhibits ccRCC progression via regulating the circDVL1/miR-412-3P/PCDH7 pathway.

We examined the expression of circDVL1, miR-412-3p, and PCDH7 in 18 sets of paired ccRCC and normal adjacent specimens by qPCR. circDVL1 and PCDH7 mRNA expression was significantly downregulated while miR-412-3p were highly expressed in ccRCC tumors relative to paired normal tissue samples (P < 0.05) (Fig. 8A-C). Interestingly, Pearson correlation analysis showed a positive association between circDVL1 and PCDH7 mRNA levels in 18 paired ccRCC samples, while both circDVL1 and PCDH7 expression were negatively correlated with miR-412-3p expression (Fig. 8D-F). These results not only demonstrate the dysregulation of circDVL1, miR-412-3p, and PCDH7 in ccRCC tissues but also suggest the existence of circDVL1/miR-412-3p/PCDH7 in human ccRCC (Fig. 8G).

## Discussion

Different types of ccRCC biomarkers have been proposed and evaluated experimentally, including mRNAs 32, miRNAs 33, lncRNAs 34, and circRNAs 35. However, none of them have been confirmed as accurate diagnostic biomarkers for routine clinical use in ccRCC patients. Here, our study illustrated that circDVL1 plays an important role in ccRCC progression. First, we screened serum from ccRCC patients and healthy controls to identify differentially expressed circRNAs using a circRNA microarray. The RT-qPCR data revealed that circDVL1 was dramatically downregulated in ccRCC samples. Previous studies have shown that circRNAs could serve as latent serum biomarkers in different cancers 36, 37. For example, Bei et al. demonstrated that exosomal hsa-circ-0004771 was increased in serum collected from colorectal cancer patients 38. Nevertheless, research on the specific role of circRNA as a non-invasive biomarker in ccRCC is still limited. Our study revealed that circDVL1 was significantly downregulated in serum from ccRCC patients and that the decreased expression of serum circDVL1 may be tumor-derived. Furthermore, serum circDVL1 level had significant diagnostic value in discriminating between patients with Fuhrman grade I-II and Fuhrman grade III-IV tumors. Therefore, serum circDVL1 could be a promising diagnostic marker for ccRCC. However, several healthy patients showed low levels of circDVL1 in their serum, indicating that patient monitoring solely based on the analysis of this circulating RNA could result in false-positive results. Therefore, the value of circDVL1 as a single diagnostic marker is somewhat limited and a combination of different circulating RNAs might need to be analyzed to obtain a reliable prediction for tumor development and metastasis.

As a newly identified circRNA, the biological and pathological role of circDVL1 had not been investigated. In this study, we evaluated the role and regulatory mechanism of circDVL1 in ccRCC. Functional assays showed that overexpressed circDVL1 could decrease ccRCC cell proliferation, colony formation ability, and tumorigenic potential, likely due to increased cell apoptosis *in vitro* and suppressed tumor development *in vivo*. The data implied that circDVL1 could have a tumor suppressor function in ccRCC.

CircRNAs can promote tumor initiation and progression by acting as miRNA sponges. Hence, we examined whether circDVL1 could regulate miRNA biogenesis during ccRCC carcinogenesis. Using miRanda and miRWalk software, we found that circDVL1 had a potential binding site for miR-412-3p. Luciferase reporter assays demonstrated a direct link between circDVL1 and miR-412-3p. Previous studies have shown that miR-412-3p was involved in the pathogenesis of different cancers through various molecular mechanisms 31, 39, 40. For example, miR-412-3p had an oncogenic role in colon cancer, promoting migration, invasion, and cell proliferation of cancer stem cells 31. Moreover, miR-412-3p expression was upregulated in extracellular vesicles from oral squamous cell carcinoma (OSCC) patients 39. Similarly, our results suggested that miR-412-3p was an important tumor promoter in ccRCC.

In addition, the target of circDVL1/miR-412-3p was also identified. Protocadherins (PCDHs) are transmembrane proteins belonging to the cadherin superfamily, which play significant roles in cell adhesion and signaling pathways. PCDH7 is a member of the PCDH family and has well-established roles in cell-cell recognition and cell adhesion 41. It is generally known that loss of adhesion is an early step in tumor cell invasion and metastasis. Emerging evidence suggests that PCDH7 is aberrantly expressed in a variety of tumor types. For example, in human non-small cell lung cancer, PCDH7 was frequently overexpressed and functioned as an oncogene to induce cellular transformation and promote lung tumorigenesis by potentiating MAPK signaling 42. Conversely, PCDH7 inhibited migration and invasion of gastric cancer cells via E-cadherin 43. Moreover, reduced PCDH7 expression has been found in colorectal 44 and bladder cancers 45. These data indicated that PCDH7 had various functions in distinct tumor types. In this study, we demonstrated that PCDH7 levels were reduced in ccRCC tumor specimens. Interestingly, functional rescue experiments revealed that the tumor suppressor properties of circDVL1 are partly due to its effect on PCDH7. Our study strongly suggests the importance of circDVL1/miR-412-3p/PCDH7 regulatory axis in ccRCC progression.

This study has several limitations. We tested three specific siRNAs for endogenous circDVL1, but unfortunately, all of these siRNAs failed to knockdown circDVL1 probably due to the unique structure of this circRNA, as well as its low basal expression in ccRCC cells. In addition, our results showed that circDVL1 could be a therapeutic target in ccRCC patients, clinical studies with large cohort of patients are required to further demonstrate its clinical importance. In our study, we did not investigate the cause of the downregulation of circDVL1 in ccRCC lesions. It has been reported that RNA-binding proteins (RBPs), including quaking (QKI), epithelial-splicing regulatory protein 1 (ESRP1), and fused-in-sarcoma (FUS) are involved in the posttranscriptional formation of circRNAs 46-48. In addition, some transcription factors (e.g., ZEB1, c-Myb, and c-Jun) are also implicated in the biogenesis of specific circRNAs 49-52. Also, studies have shown that m6A modification mediates circRNA degradation. Park et al. reported that m6A-modified circRNAs were also downregulated via a YTHDF2-HRSP12-RNase P/MRP axis 53. Whether these RBPs, transcription factors, and m6A methylation are involved in the downregulation of circDVL1 remains uncertain. In our future research, we will try to explore the upstream regulatory mechanism of circDVL1 in ccRCC development and tumorigenesis.

In summary, we demonstrated that circDVL1 level was reduced in serum from ccRCC patients. Notably, we further investigated the regulatory mechanisms of circDVL1 and demonstrated that circDVL1 suppressed ccRCC tumorigenesis by promoting PCDH7 expression by sponging miR-412-3p. Therefore, our study showed that circDVL1 might be a potential therapeutic target for future ccRCC treatments.

## Supplementary Material

Supplementary figures and tables.

## Abbreviations

RCC: renal cell carcinoma
circRNAs: circular RNAs
ncRNAs: non-coding RNAs
miRNAs: microRNAs
FISH: fluorescence in-situ hybridization
ncRNA: non-coding RNA
qRT-PCR: quantitative real-time PCR
RIP: RNA immunoprecipitation
RNA-seq: RNA sequencing
TCGA: The Cancer Genome Atlas
UTRs: untranslated regions
DAPI: 4', 6-diamidino-2-phenylindole
PCDH7: protocadherin7
IHC: immunohistochemical staining
