# Supplementary material for: Circular RNA circDVL1 inhibits clear cell renal cell carcinoma progression through the miR-412-3p/PCDH7 axis
Source: Int J Biol Sci. 2022 Jan 24;18(4):1491–507. doi: 10.7150/ijbs.69351 (PMC8898370; doi:10.7150/ijbs.69351)
Supplement: Supplementary file 1 — Supplementary figures and tables. [file ijbsv18p1491s1.pdf]

Supplementary Data

Circular RNA circDVL1 inhibits clear cell renal cell carcinoma progression through the miR-412-3p/PCDH7 axis

Ying Wang[1,2*], Yunjing Zhang[1,2*], Xinwan Su[3], Qiongzi Qiu[4], Yuan Yuan[2], Chunhua Weng[2], Sailan Zou[5], Yan Tian[5], Weidong Han[6], Pengyuan Liu[4], Xingyi Guo[7], Jianhua Mao[8], Xianghui Fu[5], Ping Wang[3], Weiqiang Lin[1]

[1]The Fourth Affiliated Hospital, and International Institutes of Medicine, Zhejiang University School of Medicine, Jinhua 322000, Zhejiang, China.

[2]Kidney Disease Center, the First Affiliated Hospital, and Institute of Translational Medicine, Zhejiang University School of Medicine, Hangzhou 310003, Zhejiang, China.

[3]Department of Urology, the First Affiliated Hospital, Zhejiang University School of Medicine, Hangzhou 310003, Zhejiang, China.

[4]Department of Respiratory Medicine, Sir Run Run Shaw Hospital and Institute of Translational Medicine, Zhejiang University School of Medicine, Hangzhou 310016, Zhejiang, China.

[5]Division of Endocrinology and Metabolism, State Key Laboratory of Biotherapy and Cancer Center, West China Hospital, Sichuan University and Collaborative Innovation Center of Biotherapy, Chengdu 610041, Sichuan, China.

[6]Department of Medical Oncology, Sir Run Run Shaw Hospital, Zhejiang University School of Medicine, Hangzhou 310016, Zhejiang, China.

[7]Division of Epidemiology, Department of Medicine, Vanderbilt Epidemiology Center, and Vanderbilt-Ingram Cancer Center, Vanderbilt University School of Medicine, Nashville 37203, TN, USA.

[8]Department of Nephrology, National Clinical Research Center For Child Health, Children's Hospital, Zhejiang University School of Medicine, Hangzhou 310003, Zhejiang, China.

Corresponding author: Dr. Weiqiang Lin, Zhejiang University School of Medicine, Email: wlin@zju.edu.cn, Tel: 86-571-86971990; or Dr. Ping Wang, Zhejiang University School of Medicine, Email: wpyyy@126.com, Tel: 86-571-87236833; or Dr. Xianghui Fu, State Key Laboratory of Biotherapy and Cancer Center, Sichuan University, Email: xfu@scu.edu.cn, Tel: 86-28-85053582.

Table S1. Correlation between circDVL1 and clinicopathologic characteristics of 60 patients with ccRCC

Variables	Overall (n=60)	circDVL1		*P*
		Low(n=34)	High(n=26)	
Age				
<60		22	17	1.00
≥60		12	9	
Gender				
Male		17	20	0.06
Female		17	6	
Fuhrman grade				
I/II		24	24	0.05
III/IV		10	2	

Table S2 primers information

Gene	Primer sequences
β -actin	F: 5'- ACTCTTCCAGCCTTCCTTCC-3'<br>R: 5'- CGTCATACTCCTGCTTGCTG-3'
circDVL1	F: 5'-ATCTCTGCAGCACTGACCCG-3'<br>R: 5'-ACGGCGCTCATGTCACTCTT-3'
DVL1	F: 5'- GAGGGTGCTCACTCGGATG-3'<br>R: 5'- GTGCCTGTCTCGTTGTCCA-3'
circ_00063152	F: 5'- ACAAGGGCAAGAAGAGGCAC-3'<br>R: 5'- CCGCCCTCCTTGCATCATAC-3'
circ_0030264	F: 5'- GTGTGAACCAGAGATGTCGGC-3'<br>R: 5'- CGCGATCTCTTGAGGGGAGA-3'
circ_0000118	F: 5'- TGGGCAAAGATGGATTGAAGACA-3'<br>R: 5'- TCCTCTTCCCTCCTCTCCTGAT-3'
circ_0109946	F: 5'- CCCACACACCTTACATTCGTACAT-3'

	R: 5'- AAGTGTGTGAGGAAACCTTTAGGC-3'
circ_0081215	F: 5'- CCATTGTTGGTTGGGGTGCT-3'
	R: 5'- CGTCGCTTCCTCAAGGCAAA-3'
CDK6	F: 5'- TCTTCATTCACACCGAGTAGTGC-3'
	R: 5'- TGAGGTTAGAGCCATCTGGAAA-3'
GAPDH	F: 5'- GAAGGTGAAGGTCGGAGTC-3'
	R: 5'- GAAGATGGTGATGGGATTTC-3'
DAG1	F: 5'-AGCAAAGGATTGACCTCCTGC-3'
	R: 5'-CCACCGGCACTAATTTCATGTT-3'
SEMA4D	F: 5'-CGCCACTTCCCTTTACGTGT-3'
	R: 5'-ACCATGACGGATGTGTAGCTG-3'
CHST11	F: 5'-AGAAATCAACCACCGCTTGAA-3'
	R: 5'-GTAGGCGGACACTAGCCTC-3'
ZBTB20	F: 5'-GACAGGATCTACTCGGCACTC-3'
	R: 5'-ACTGCGCCGCTGTAAAAAGA-3'
PTPN14	F: 5'-GGACAAATTCGCTAATGAGCCT-3'
	R: 5'-TAATCGCCCTTCAAGCACATC-3'
PCDH7	F: 5'-TCAACGGGCAGATCGAATACG-3'
	R: 5'-TCTTGCGGATTTCAATGGACG-3'
SMAD3	F: 5'-TGGACGCAGGTTCTCCAAAC-3'
	R: 5'-CCGGCTCGCAGTAGGTAAC-3'
PPM1H	F: 5'-AGGCTGGGCATACAAAACCAT-3'
	R: 5'-CACTCCAATAGTTGCCATTACCC-3'
miR-412-3p	F: 5'-TACTTCACCTGGTCCACTAGCCGTC-3'

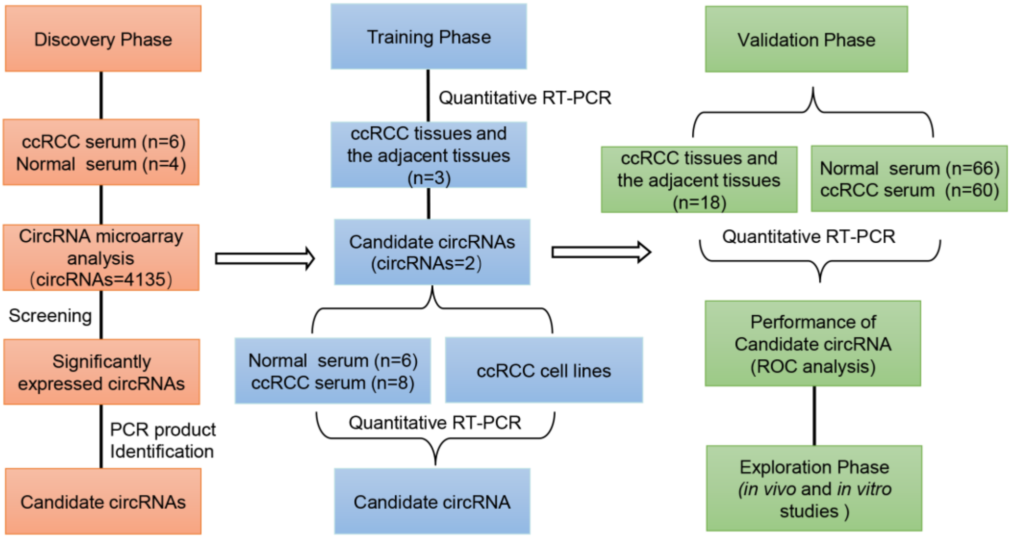

Figure S1 Study design.

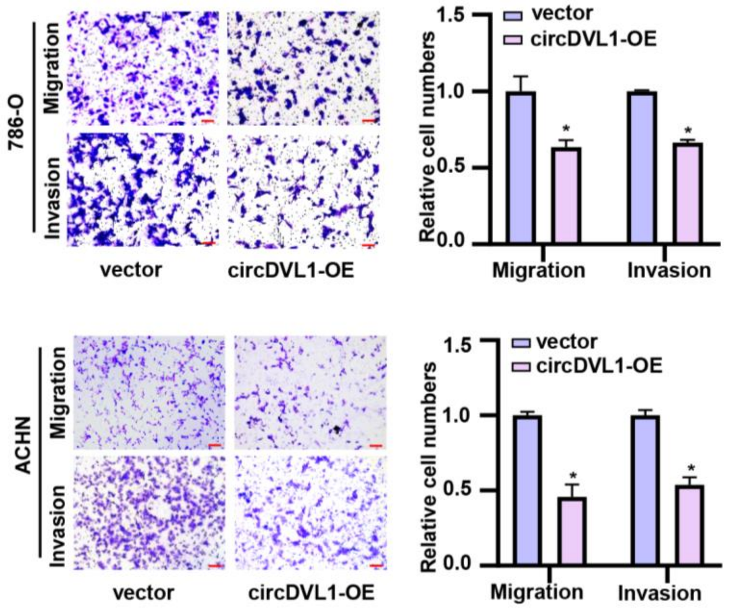

Figure S2 Transwell assays revealed that circDVL1 overexpression suppressed the migration and invasion capacities of 786-O and ACHN cells. circDVL1-OE, circDVL1 overexpression. vector, negative control for overexpression. Scale bar, 100 μm. Data are exhibited as mean ± SD. *$P < 0.05$, ** $P < 0.01$, *** $P < 0.001$, **** $P < 0.0001$, no significant (NS).

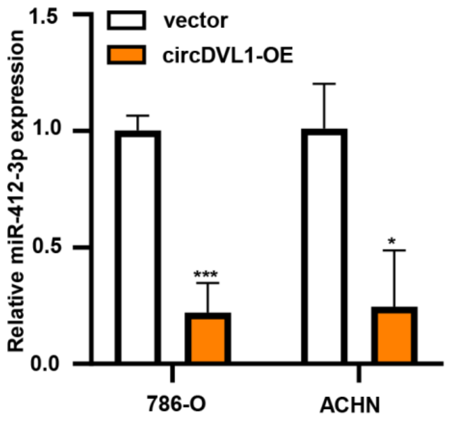

Figure S3 Relative expression of miR-412-3p was detected in cells after transfection with control vector and circDVL1 overexpression vector using qRT-PCR. Data are presented as means ± SD. *P < 0.05, ** P < 0.01, *** P < 0.001, **** P < 0.0001, not significant (NS).

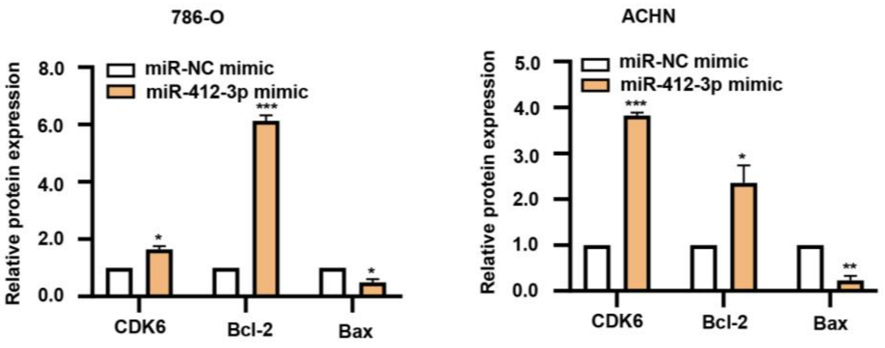

Figure S4 Quantification of western blot results of CDK6, Bcl-2, and Bax protein levels in 786-O and ACHN cells after transfection with miR-412-3p mimic or miR-NC. Data are presented as means ± SD. *P < 0.05, ** P < 0.01, *** P < 0.001, **** P < 0.0001, not significant (NS).

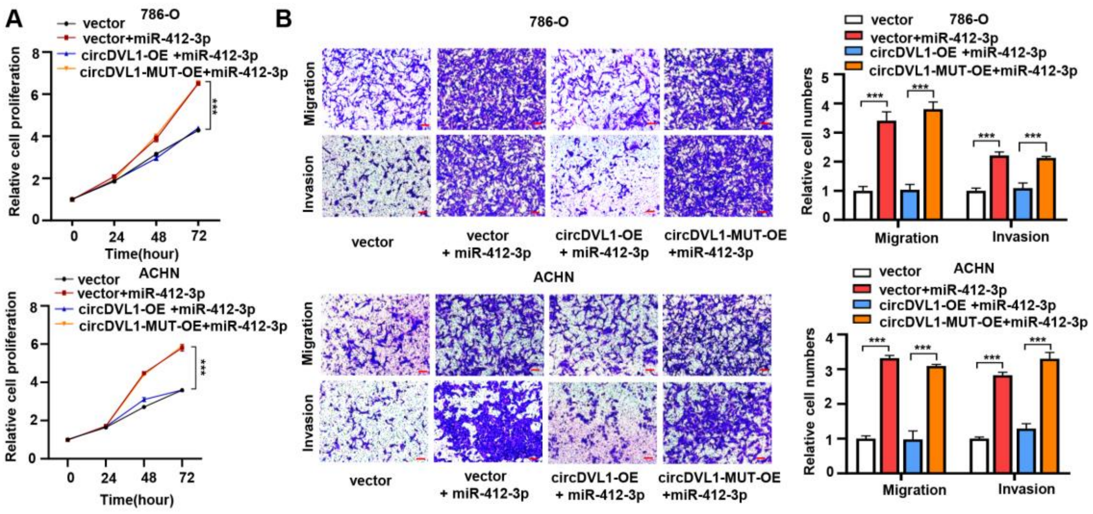

Figure S5 (A) CCK-8 cell proliferation analysis of 786-O and ACHN transfected with control vectors, vector+miR-412-3p, miR-412-3p+circDVL1-OE, miR-412-3p+ circDVL1-MUT-OE. (B) Transwell cell invasion and migration assay in treated 786-O and ACHN cells. Scale bar, 100 μm. circDVL1-OE, circDVL1 overexpression vector, negative control for overexpression, circDVL1-MUT-OE, overexpression of circDVL1 mutation vector. Data are presented as means ± SD. *$P < 0.05$, ** $P < 0.01$, *** $P < 0.001$, **** $P < 0.0001$, not significant (NS).

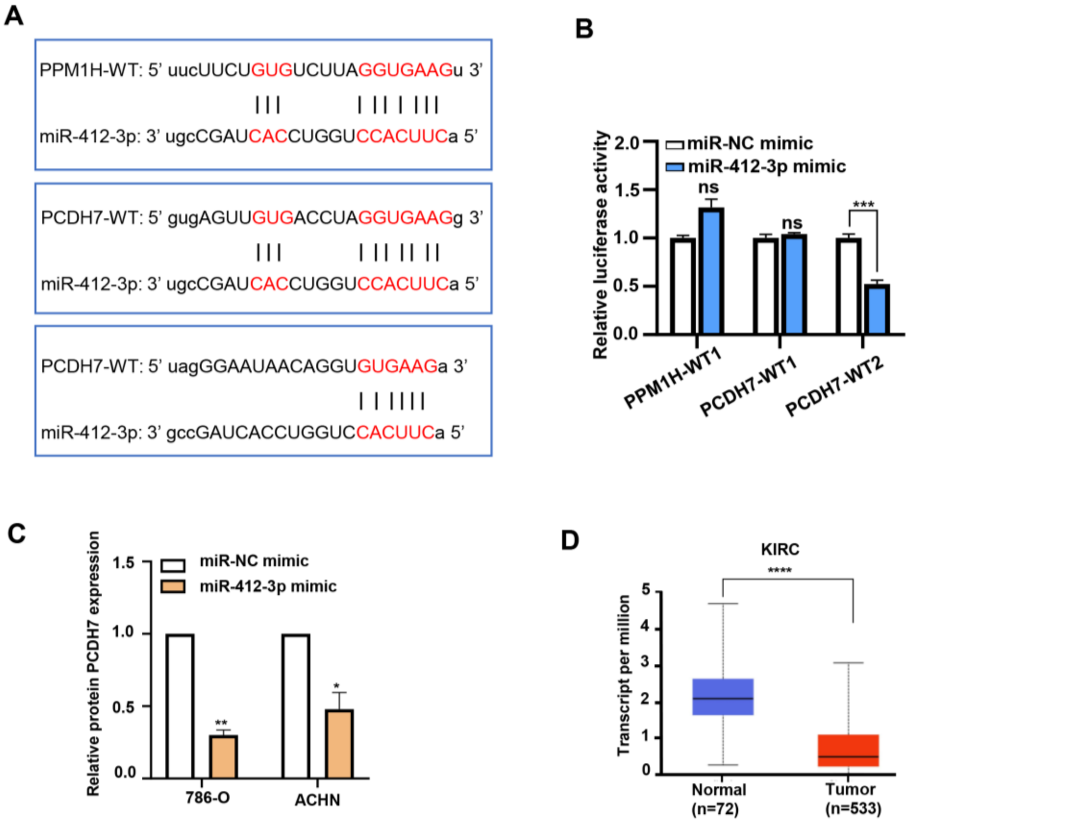

Figure S6 (A) WT sequence of the putative binding sites in miR-412-3p, PPM1H, and PCDH7, respectively. (B) Luciferase activities were measured in 293T cells after transfection with PPM1H-WT, PCDH7-WT1, or PCDH7-WT2 and miR-412-3p or miR-NC mimics. (C) Western blot results of PCDH7 protein levels in ccRCC cells after transfection with miR-412-3p mimic or miR-NC. (D) Box-plot of PCDH7 expression level in TCGA ccRCC tumors and matched normal tissue samples. Data are presented as means ± SD. *P < 0.05, ** P < 0.01, *** P < 0.001, **** P < 0.0001, not significant (NS).

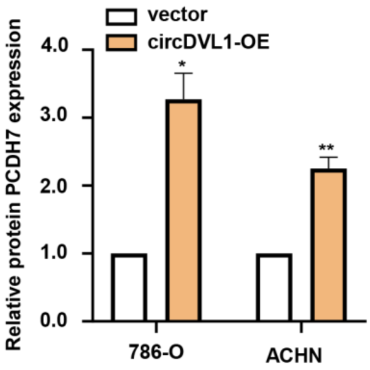

Figure S7 Quantification of western blot results of PCDH7 protein levels in ccRCC cells after transfection with control vector and circDVL1 overexpression vector. Data are presented as means ± SD. *P < 0.05, ** P < 0.01, *** P < 0.001, **** P < 0.0001, not significant (NS).

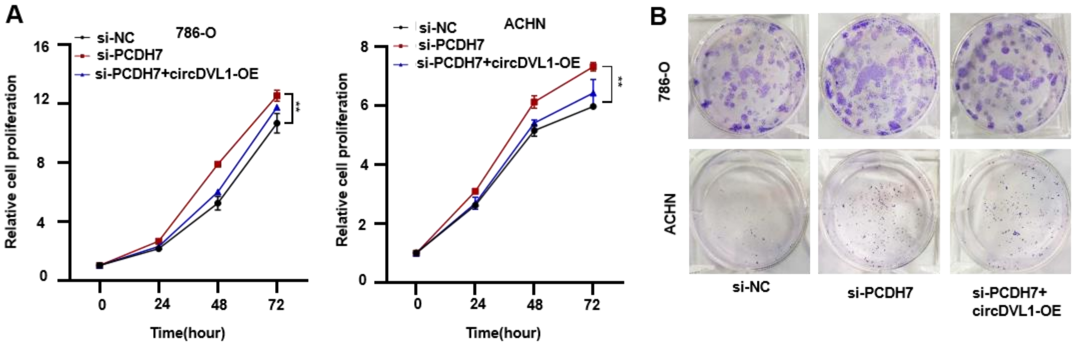

Figure S8 (A) and (B) Silencing of PCDH7 can suppress the inhibition of cell proliferation and colony formation capacity in circDVL1 overexpressing cells. Data

are presented as means ± SD. *$P < 0.05$, ** $P < 0.01$, *** $P < 0.001$, **** $P < 0.0001$, not significant (NS).
